# Repositioning the Role of Tumor Necrosis Factor-Related Apoptosis-Inducing Ligand (TRAIL) on the TRAIL to the Development of Diabetes Mellitus: An Update of Experimental and Clinical Evidence

**DOI:** 10.3390/ijms23063225

**Published:** 2022-03-17

**Authors:** Chrysi Koliaki, Nicholas Katsilambros

**Affiliations:** First Propaedeutic Department of Internal Medicine and Diabetes Center, Medical Faculty, Laiko General Hospital, National Kapodistrian University of Athens, 17 Agiou Thoma Street, 11527 Athens, Greece; nicholaskatsilambros@gmail.com

**Keywords:** tumor necrosis factor-related apoptosis-inducing ligand (TRAIL), TRAIL receptors, type 1 diabetes mellitus (T1DM), type 2 diabetes mellitus (T2DM), obesity, insulin resistance

## Abstract

Tumor necrosis factor (TNF)-related apoptosis-inducing ligand (TRAIL), a member of the TNF protein superfamily, represents a multifaceted cytokine with unique biological features including both proapoptotic and pro-survival effects in different cell types depending on receptor interactions and local stimuli. Beyond its extensively studied anti-tumor and immunomodulatory properties, a growing body of experimental and clinical evidence over the past two decades suggests a protective role of TRAIL in the development of type 1 (T1DM) and type 2 (T2DM) diabetes mellitus. This evidence can be briefly summarized by the following observations: (i) acceleration and exacerbation of T1DM and T2DM by TRAIL blockade or genetic deficiency in animal models, (ii) prevention and amelioration of T1DM and T2DM with recombinant TRAIL treatment or systemic TRAIL gene delivery in animal models, (iii) significantly reduced circulating soluble TRAIL levels in patients with T1DM and T2DM both at disease onset and in more advanced stages of diabetes-related complications such as cardiovascular disease and diabetic nephropathy, (iv) increase of serum TRAIL levels in diabetic patients after initiation of antidiabetic treatment and metabolic improvement. To explore the underlying mechanisms and provide mechanistic links between TRAIL and diabetes, a number of animal and in vitro studies have reported direct effects of TRAIL on several tissues involved in diabetes pathophysiology such as pancreatic islets, skeletal muscle, adipose tissue, liver, kidney, and immune and vascular cells. Residual controversy remains regarding the effects of TRAIL on adipose tissue homeostasis. Although the existing evidence is encouraging and paves the way for investigating TRAIL-related interventions in diabetic patients with cardiometabolic abnormalities, caution is warranted in the extrapolation of animal and in vitro data to the clinical setting, and further research in humans is imperative in order to uncover all aspects of the TRAIL-diabetes relationship and delineate its therapeutic implications in metabolic disease.

## 1. Introduction

Tumor necrosis factor (TNF)-related apoptosis-inducing ligand (TRAIL) belongs to the TNF superfamily of proteins. The TNF superfamily of ligands and their receptors play an important role in regulating multiple fundamental cellular processes such as cell death and survival, proliferation, differentiation and immune surveillance [1]. The first characterized biological function of TRAIL was its ability to induce apoptosis in cancer cells and regulate host defense against tumor initiation and progression [2]. Beyond the originally described anti-tumor effects, TRAIL has demonstrated important immunomodulatory properties and is considered to act as a valuable safeguard against malignant and autoimmune diseases maintaining immune homeostasis [3,4]. Its unique biological feature, compared to other TNF superfamily members such as CD95L (Fas Ligand; FasL) and TNF-α, is the ability to selectively induce apoptosis in most transformed cells such as the malignant or virally infected ones, while preserving normal (non-transformed) cells [5,6]. TRAIL can activate not only apoptosis, but also pathways promoting cell survival and proliferation [7,8,9,10]. Of note, the ultimate biologic outcome of TRAIL stimulation is determined in a cell type-specific context by the coordinated interaction of various different elements, comprising—but not restricted to—the expression, localization, and redistribution of membrane receptors and other critical intracellular components that can switch cell phenotypes between proapoptotic and anti-apoptotic ones [10,11].

Over the last two decades, several research groups have addressed the pleiotropic roles of TRAIL not only in malignancy but also in other diseases. There has been an increased interest in exploiting the potential of TRAIL to treat metabolic diseases [12,13], based on a growing body of experimental and clinical evidence suggesting that the TRAIL system plays an important role in the development and progression of both autoimmune (T1DM) and obesity-associated (T2DM) diabetes mellitus [13,14]. The association of TRAIL with diabetes becomes evident through a number of findings provided by animal and human studies, which briefly comprise the following: (i) the onset and severity of T1DM and T2DM can be accelerated and exacerbated by TRAIL blockade or TRAIL genetic deficiency [15,16,17], (ii) T1DM and T2DM can be effectively prevented and ameliorated by recombinant TRAIL treatment or systemic TRAIL gene delivery [18,19,20,21,22], (iii) circulating soluble TRAIL levels are significantly reduced in patients with T1DM, T2DM and diabetes-related macro- and microvascular complications [23,24,25,26,27,28,29], (iv) serum TRAIL levels of patients with T2DM progressively increase upon antidiabetic treatment [30]. To explore the underlying mechanisms accounting for the TRAIL-diabetes relationship, several in vitro studies have been conducted and report direct effects of TRAIL on several tissues involved in diabetes pathophysiology, such as pancreatic islets, skeletal muscle, adipose tissue, liver, kidney, and immune and vascular cells [7,20,31,32,33,34].

The scope of the present narrative review was to critically summarize the most recent experimental and clinical findings regarding the role of TRAIL in T1DM and T2DM, discuss possible underlying mechanisms for the protective role of TRAIL in T1DM and T2DM by focusing on tissue-specific effects, provide an update for the role of TRAIL in important diabetes-related complications such as ischemic heart disease and diabetic nephropathy, and highlight controversies and open questions in the field of TRAIL and metabolic disease.

## 2. Literature Search and Review Criteria

The studies selected for this review were identified by a computer search program using PubMed electronic database for scientific literature published in English until February 2022. The following search terms were applied: “TRAIL type 1 diabetes mellitus”, “TRAIL type 2 diabetes mellitus”, “TRAIL obesity”, “TRAIL insulin resistance”, “TRAIL diabetes complications”, “TRAIL diabetic nephropathy”, and “TRAIL diabetes cardiovascular disease”. Additional references were retrieved from reviewing the references cited in the original articles. Our literature search included both animal and human studies. Mechanistic in vitro studies were also included to elucidate the pathophysiological traits of the relationship between TRAIL biology and diabetes in different tissues.

## 3. Brief Overview of TRAIL Biology and Signaling Pathways

Since its discovery in 1995 [5], TRAIL (also known as TNF superfamily member 10; TNF-SF10) has been recognized as a cytokine with pleiotropic biological effects in multiple different cell types. Human TRAIL is a 281 amino acid type II transmembrane protein whose molecular structure is characterized by the presence of a TNF homology domain, which binds to cysteine-rich regions of specific receptors [1]. Endopeptidases can release soluble TRAIL, which is the biologically active ligand, by proteolytic cleavage of its membrane-bound form [1]. After cleavage, soluble TRAIL assembles with two other molecules of TRAIL to form a trimeric ligand, and TRAIL homotrimers bind with their specific receptors [35]. Unlike FasL or TNF-α, which have one or two functional receptors, soluble TRAIL interacts with four different transmembrane and one soluble receptor, demonstrating the impressive biological complexity of this ligand.

**Figure 1:**
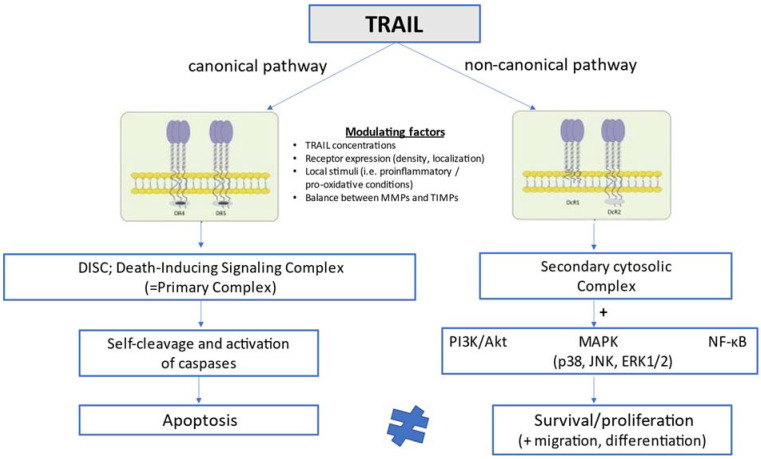
Schematic presentation of TRAIL signaling pathways at the cellular level.

In humans, TRAIL is implicated in the pathway of extrinsic (death ligand-mediated, mitochondria-independent) apoptosis by engaging with two death-inducing receptors, TRAIL-R1 [death receptor 4 (DR4)] and TRAIL-R2 [death receptor 5 (DR5)], which have functional intracellular death domains that result ultimately in apoptosis through a pathway dependent on sequential caspase activation [36,37,38,39]. TRAIL may also interact with three other receptors, the decoy receptors DcR1 (TRAIL-R3) and DcR2 (TRAIL-R4), as well as the soluble decoy receptor osteoprotegerin (OPG; TRAIL-R5), which contain non-functional cytoplasmic death domains and fail thus to mediate apoptotic signaling [40,41,42]. The functions of these decoy or else regulatory receptors remain poorly understood, but they are considered to act by sequestering ligands in a competitive manner and thereby counteracting and attenuating proapoptotic signals [37,42]. In mice, only one receptor, DR5, has been described to share 60% degree of structural homology with human TRAIL-R2 [43]. TRAIL and its receptors are expressed in a variety of cell types, including T-cells, B-cells, macrophages, natural killer (NK) cells, and dendritic cells [44,45,46], and its expression is significantly upregulated in activated immune cells upon cytokine stimulation [47]. The lack of toxicity of TRAIL in most normal cells under physiological conditions and its ability to induce opposite cellular responses (apoptosis vs. survival/proliferation) in different cells has been related to the high number of receptors that can mediate TRAIL signaling in a differential manner, local conditions such as proinflammatory and pro-oxidative states which can either facilitate or inhibit TRAIL signal transduction, and the interaction with other circulating TNF-related cytokines such as OPG [48]. As schematically presented in Figure 1, TRAIL can activate not only extrinsic apoptosis and other pathways of cell death such as necroptosis [49] and autophagy [9,50], but also opposing non-apoptotic cellular signaling pathways promoting survival, proliferation, migration and differentiation [51,52]. The latter pathways (non-canonical TRAIL signaling) go along with phosphorylated kinase and proinflammatory pathways, and are mainly mediated by PI3K/Akt, mitogen-activated protein kinase (MAPK) signaling, which includes p38, c-Jun N-terminal kinase (JNK), and extracellular signal-regulated kinases 1 and 2 (ERK1/ERK2), as well as the transcription factor nuclear factor kappa B (NF-κB) [8,42].

The versatile biology of TRAIL has been related not only to its complex receptor system and its potential modulation by local stimuli, but also to other factors, such as the exposure of membrane receptors to different TRAIL concentrations [11] and the balance between metalloproteinases and tissue inhibitors of metalloproteinases, which is thought to be involved in the clearance of circulating TRAIL [53].

In the canonical pathway, TRAIL stimulates extrinsic apoptosis by binding to the two membrane death receptors TRAIL-R1 (DR4) and TRAIL-R2 (DR5), leading to the formation of DISC or else the primary complex. This complex triggers in turn a cascade of sequential self-cleavage and activation of caspases, leading ultimately to apoptosis. In the non-canonical pathway, TRAIL interacts with the decoy receptors TRAIL-R3 (DcR1) and TRAIL-R4 (DcR2), which lack functional intracellular death domains, and promotes the formation of the secondary cytosolic complex alternatively to DISC. The secondary complex activates in turn a number of pathways related to cell survival and proliferation, such as pathways mediated by PI3K/Akt, MAPK (p38, JNK, ERK1/2), and NF-κB. Whether TRAIL will preferentially stimulate either the canonical (proapoptotic) or the non-canonical (anti-apoptotic) pathway is determined by a number of interfering modulating factors comprising TRAIL circulating concentrations, TRAIL receptor expression, density and localization, local stimuli such as proinflammatory and pro-oxidative conditions, and the balance between matrix metalloproteinases and their tissue inhibitors, which is considered to affect the clearance of circulating TRAIL.

## 4. The Role of TRAIL in T1DM

Table 1 summarizes the most representative experimental and clinical studies investigating the relationship of TRAIL with T1DM in animals and humans [15,18,21,22,23,31,54].

A number of sophisticated experimental studies from as long as 20 years ago have used mouse models of autoimmune diabetes to explore the role of TRAIL in the pathogenesis of T1DM [15,31]. Two commonly studied animal models sharing several clinical and histological resemblances with human T1DM include non-obese diabetic (NOD) mice treated with cyclophosphamide to accelerate diabetes onset, and C57BL/6 mice treated sequentially with multiple low doses of streptozotocin to induce pancreatic inflammatory damage [55]. It has been shown that TRAIL gene and protein is overexpressed in pancreatic islets of mice during the development of autoimmune diabetes [31], in pancreatic sections of children with acute-onset fatal T1DM [56], and in β-cell cultures upon cytokine activation [31]. In NOD mice, the exact role of TRAIL has been examined using a soluble TRAIL receptor (sDR5) to inhibit the endogenous TRAIL biological activity. TRAIL functional blockade by systemic administration of sDR5 was shown to exacerbate cyclophosphamide-induced diabetes in NOD mice, augment the severity of pancreatic islet inflammation (insulitis), and enhance the T1DM-specific immune response driven by anti-GAD65 autoantibodies [15]. In the second model of streptozotocin-induced diabetes, TRAIL genetic deficiency (knockout) was found to increase the incidence of diabetes and aggravate the histological pattern of islet inflammation [15]. The above data clearly suggest that a defective TRAIL function may upregulate pancreatic autoimmune responses by enhancing autoreactive T-cell activation, and act thereby as an accelerator for the development of T1DM. It has been also intriguingly proposed that islet cells may have a self-defense system which can control their survival by locally regulating autoimmune reactions mediated by TRAIL, considering that TRAIL expression is upregulated in cytokine-activated or apoptotic pancreatic islets [31]. This initial stimulation of TRAIL expression and activity during the course of destructive insulitis has been suggested as a potential protective mechanism to counteract or even delay the onset of the disease. However, with sustained insult and in the absence of additional interventions, progression into overt diabetes may occur.

In agreement with the above studies revealing a detrimental role of TRAIL depletion for T1DM development, additional experimental studies using either recombinant TRAIL treatment [18] or systemic adenoviral vector-mediated TRAIL gene therapy [21,22] could further substantiate the protective role of TRAIL signaling for the prevention and treatment of T1DM. In this context, recombinant TRAIL treatment has been shown to ameliorate streptozotocin-induced diabetes in mice by reducing hyperglycemia, preventing catabolic manifestations such as weight loss, and partially preserving pancreatic islet morphology and residual insulin secretion [18]. These beneficial effects of TRAIL treatment in terms of alleviating the metabolic and histological manifestations of autoimmune diabetes were associated with a significant anti-inflammatory activity, as shown by the reduced expression of cytokines involved in both systemic (TNF-α and OPG) and pancreatic (vascular cellular adhesion molecule-1; VCAM-1) inflammation [18]. With regard to the second intervention leading to TRAIL effect augmentation, namely systemic TRAIL gene delivery via an adenovirus, this approach has been shown to prevent T1DM in NOD mice [21]. This effect was mediated by an enhanced inhibition of pancreatic matrix metalloproteinases (MMPs), which is thought to suppress the transmigration of diabetogenic T-cells into pancreatic islets and further protect β-cells from cytokine-induced apoptosis [21]. Furthermore, it has been shown that adenovirus-mediated TRAIL gene delivery into pancreatic islets was associated with sustained normoglycemia in streptozotocin-induced diabetic rats compared with animals grafted with mock-infected islets [22].

In humans, it has been demonstrated that the serum circulating levels of TRAIL are significantly reduced in patients with T1DM, with the lowest levels observed in those presenting with ketoacidosis at onset and those with the highest insulin requirements, reflecting an advanced severity of the underlying disease [23]. In detail, a retrospective Italian study conducted in a pediatric cohort of children with T1DM, reported a significant decrease in circulating TRAIL levels in patients with new-onset T1DM having ketoacidosis as initial presentation, compared to healthy individuals with or without islet-specific autoantibodies, and also found an inverse correlation between serum TRAIL levels at diagnosis and insulin needs up to 2 years of follow-up [23]. Of note, the reduction of circulating TRAIL levels persisted even after disease onset, namely at least one year after diagnosis. These clinical data suggest that circulating soluble TRAIL levels are significantly affected by the presence of T1DM, both at and after disease onset. Although the profound suppression of circulating TRAIL levels in T1DM patients with ketoacidosis and increased daily insulin requirements indicates a possible association between TRAIL and the severity of autoimmune reaction, not all findings of this study support this hypothesis, since there was no difference in TRAIL levels between T1DM patients with and without islet-specific antibodies, and there was no difference between diabetic patients with and without other autoimmune diseases which often coexist with T1DM [23]. It remains also unclear whether the observed reduction in circulating TRAIL levels in patients with T1DM is the result of reduced production/secretion or increased consumption at sites of inflammation. Considering that TRAIL expression is significantly upregulated in the pancreatic islets of animals with autoimmune diabetes, the theory of enhanced consumption accounting for the reduced circulating TRAIL concentrations seems most likely. Although the above study provides interesting data for the role of TRAIL in human T1DM, especially in light of the relative paucity of related clinical studies, it is limited by its retrospective design, which precludes any causal inferences regarding the modulation of TRAIL system in the setting of human T1DM. Further limitations of this study include the lack of serial blood samples from the same patients at different time points after ketoacidosis and the lack of information about the metabolic status at each time point of blood sampling. These limitations were addressed in a subsequent pilot study of the same group, which reported a significant increase of serum TRAIL levels shortly after the initiation of insulin treatment in newly diagnosed pediatric patients with T1DM and ketoacidosis, and also a strong inverse correlation between circulating TRAIL levels and the degree of metabolic decompensation assessed by blood gas analysis [54].

## 5. The Role of TRAIL in T2DM

Table 2 summarizes the major experimental and clinical studies investigating the relationship of TRAIL with T2DM in animals and humans [16,17,19,20,24,27,28,30,33,57].

In animal models of high fat diet (HFD)-induced insulin resistance, which is a hallmark of T2DM pathophysiology, TRAIL genetic deficiency was found to exacerbate insulin resistance and aspects of non-alcoholic fatty liver disease (NAFLD) such as hepatic steatosis, inflammation and fibrosis, generating the hypothesis that increasing TRAIL levels might represent an appealing therapeutic approach to improve glucose metabolism and liver histology in diabetic patients [16]. In the same mouse model, systemic TRAIL administration by weekly intraperitoneal injections has been shown to prevent and ameliorate the metabolic perturbations associated with obesity and T2DM, by reducing diet-induced adiposity, reducing hyperglycemia and hyperinsulinemia, improving peripheral insulin sensitivity, enhancing skeletal muscle free fatty acid (FFA) oxidation, reducing the expression of proinflammatory cytokines, and positively modulating adipose tissue gene expression in the direction of anti-adipogenic and anti-inflammatory effects [19]. Of note, the ability of systemic TRAIL treatment to induce beneficial cardiometabolic adaptations persisted even after the onset of T2DM disease. As shown by the same group in HFD-fed mice becoming obese and profoundly dysmetabolic after 4 weeks of HFD, TRAIL treatment proved to be effective in reversing a broad spectrum of already established metabolic abnormalities associated with T2DM [20]. More analytically, TRAIL treatment was able to reduce body weight, adipocyte hypertrophy, lipotoxicity and systemic inflammation, restore abnormal glucose metabolism, and interestingly, improve multiple features of NAFLD by significantly reducing liver fat content and upregulating the hepatic expression of peroxisome proliferator-activated receptor γ co-activator-1α (PGC-1α) involved in mitochondrial biogenesis both in vivo and in vitro [20]. The above data, combined, corroborate the potential of TRAIL to improve not only glucose homeostasis but also a broad spectrum of obesity- and T2DM-associated derangements, both at early and more advanced stages of cardiometabolic disease.

Although these animal data reveal a beneficial impact of TRAIL upon adipose tissue homeostasis and suggest a protective effect against obesity which is tightly linked with T2DM, a number of other experimental studies investigating the direct metabolic effects of TRAIL on fat cell biology in vitro provide controversial evidence suggesting a rather obesogenic effect by promoting an insulin-resistant, inflammatory and dysfunctional phenotype of adipose tissue [58,59,60]. It has been shown that TRAIL can regulate human adipocyte metabolism by interfering with TRAIL-R2 (DR5) and inducing a caspase-mediated cleavage and thus inactivation of peroxisome proliferator-activated receptor γ (PPAR-γ), leading to suppressed de novo lipogenesis and reduced insulin-stimulated glucose uptake reflecting insulin resistance at the level of adipose tissue [58]. The mediators of TRAIL action in human adipocytes were found to be caspases, and not the NF-κB or insulin-stimulated kinase (PI3K/Akt) pathways [58]. In the same direction, TRAIL has been found to inhibit human adipocyte differentiation through a caspase-mediated down-regulation of adipogenic transcription factors [61]. It has been further shown in vitro that TRAIL triggers an inflammatory response in human adipocytes via NF-κB and ERK1/2-mediated pathways by stimulating the expression of multiple chemokines and cytokines in a dose-dependent manner [59]. Additional animal data suggest that DR5-knockout mice fed a HFD display reduced adipose tissue inflammation compared to control mice, further pointing to a proinflammatory role of TRAIL in adipose tissue [62].

On the other hand, it has been shown that TRAIL acts as a potent mitogen for adipose tissue-resident precursor cells and stimulates the proliferation of human preadipocytes by activating ERK1/2 [63]. By increasing the number of adipose progenitor cells being able to differentiate into mature adipocytes, TRAIL may contribute to the expandability of adipose tissue, which has been recognized as an important determinant of metabolic health and flexibility [64]. A possible explanation for the apparent controversy generated by the above data, which shows both positive and negative effects of TRAIL on adipose tissue function, could be provided by the hypothesis that TRAIL might exert differential effects on adipose tissue in the lean and obese state [63]. In the lean state, TRAIL appears to be expressed predominantly by fat cells, and may contribute to a healthy adipose tissue phenotype by positively affecting the preadipocyte pool and enhancing adipose tissue expandability [63]. On the other hand, it has been hypothesized that TRAIL is expressed not only by adipocytes in the obese state, but also by infiltrating macrophages and microvascular structures leading to considerably high levels of TRAIL expression within adipose tissue in the setting of obesity. Under these conditions of TRAIL excess, TRAIL might not only affect the pool of adipose stem cells, but also exert additional effects on adipocytes by modulating their metabolic functions promoting inflammation and insulin resistance [58]. Detailed studies quantifying TRAIL expression in different cell subpopulations of adipose tissue would be required to test this theory. Other possible explanations for the conflicting data on the effects of TRAIL on adipose tissue may be related to the experimental models of obesity studied, issues of species- and tissue-specificity, as well as the limited capacity of in vitro experiments to capture the complex crosstalk of adipose tissue with other tissues and reflect the in vivo physiology.

Moving to humans, clinical studies in patients with T2DM have shown that circulating serum TRAIL levels are reduced in newly-diagnosed T2DM patients [24], and they progressively increase after 6 months of antidiabetic treatment but remain still lower compared to non-diabetic control subjects even after treatment [30]. Of note, the absolute change in serum TRAIL levels has been related to the absolute change in fasting and postprandial glucose levels as well as the absolute change in glycated hemoglobin (HbA1c) before and after glucose-lowering treatment [30]. The association of TRAIL with markers of glycemic control in T2DM patients remains unclear, since some studies have shown an inverse correlation between serum TRAIL levels and HbA1c [57], while other studies in smaller cohorts did not confirm this finding [27]. With regard to human obesity, which is closely associated with T2DM, serum TRAIL levels have been positively correlated with anthropometric measures of total and central adiposity and serum lipid levels [65,66,67]. A similar correlation has been reported in patients with T2DM [68]. Since both the cellular source of circulating TRAIL and the mechanisms regulating TRAIL secretion are not completely understood, it remains unclear whether the observed positive correlation of TRAIL levels with obesity reflects an enhanced production from adipocytes or the consequence of other biological pathways activated in obesity and hyperlipidemia. Based on the anti-inflammatory properties of TRAIL, it can be assumed that higher levels of soluble TRAIL in obesity might represent an adaptive mechanism to counteract the inflammatory burden associated with obesity.

## 6. Proposed Mechanisms Underlying the Protective Role of TRAIL in T1DM and T2DM

*T1DM:* In animal models of autoimmune diseases such as T1DM, TRAIL has been shown to inhibit the proliferation of autoreactive T-cells by suppressing interleukin-2 (IL-2), IL-4 and interferon-γ (INF-γ) production, blocking DNA synthesis, preventing cell cycle progression from G1 to S phase and inhibiting calcium influx, rather than directly inducing apoptosis of activated autoantigen-specific T-cells [31,69,70]. In addition to inducing peripheral tolerance, TRAIL may also play a role in the negative selection of autoreactive T-cells in the thymus [71,72], thus regulating central tolerance as well. Of note, recent evidence suggests that TRAIL expression in regulatory T-cells (Treg) is not required for the induction of peripheral tolerance in the setting of autoimmune diabetes [73], contrary to previous data demonstrating a dual protective effect of TRAIL against autoimmunity involving both the inhibition of autoreactive T-cell proliferation and the promotion of Treg expansion [74].

Additional mechanisms implicated in the protective role of TRAIL in T1DM beyond the direct effects on immune cells infiltrating pancreatic islets involve the suppression of proinflammatory cytokine signaling by means of upregulating the expression of the immunoregulatory gene SOCS1 (suppressor of cytokine signaling 1) in pancreatic islets [18], the inhibition of pancreatic matrix degradation mediated by the elevated expression of TIMP-1 (tissue inhibitor of metalloproteinase 1), which is thought to exert antidiabetic effects by preventing MMP-mediated insulin cleavage and cytokine-induced insulitis [21], the inhibition of β-cell apoptosis (TRAIL resistance in pancreatic islets) [75], and finally the stimulation of proliferation of pancreatic β-cells through Akt activation [32], as recently shown in in vitro experiments.

*T2DM:* The beneficial effects of TRAIL in the setting of T2DM have been mainly ascribed to its immunosuppressive and immunoregulatory properties, which counteract inflammation which is a cardinal feature of T2DM [76], proliferative effects on pancreatic functional β-cell mass [32], insulin-sensitizing and myogenic effects on skeletal muscle tissue [33], and protective effects on liver consisting mainly in NAFLD amelioration [20].

The direct metabolic effects of TRAIL on skeletal muscle have been recently described in HFD-fed and genetically obese mice both in vivo and in vitro [33], and highlight the potential of TRAIL to affect whole-body metabolism through effects on skeletal muscle, which is a highly metabolically active tissue and a major determinant of systemic insulin sensitivity. In vitro, TRAIL was found to increase markers of muscle differentiation (myogenesis) and enhance insulin-mediated glucose uptake [33]. In vivo, TRAIL treatment preserved myofiber size in obese animals and was associated with an elevated skeletal muscle PGC-1α expression indicating an improved mitochondrial function and oxidative metabolism [33]. The above effects were mediated by DR5 and an increased Akt phosphorylation [33]. These data imply that TRAIL might hold important therapeutic implications not only for diabetes but also for sarcopenia, which is often observed in patients with diabetes and accounts for aging-related insulin resistance [77].

**Figure 2:**
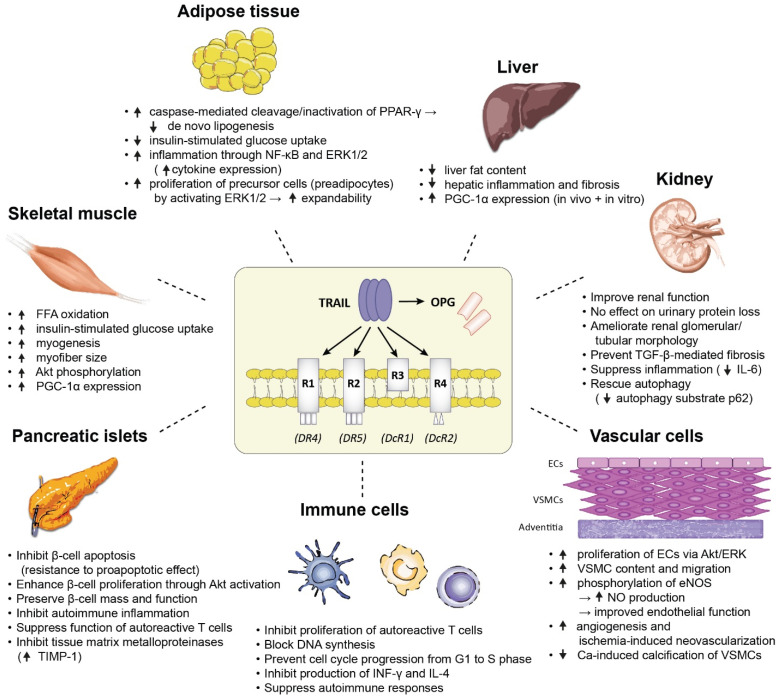
A summary of the major biological effects of TRAIL on a variety of tissues which are pathogenetically involved in the development of diabetes, as demonstrated in animal and in vitro mechanistic studies. *Abbreviations:* Akt: protein kinase B; ECs: endothelial cells; eNOS: endothelial nitric oxide synthase; ERK1/2: extracellular signal regulated kinases 1 and 2; FFA: free fatty acid; IL-4: interleukin-4; IL-6: interleukin-6; INF-γ: interferon-γ: NF-κB: nuclear factor kappa B; NO: nitric oxide; PGC-1α: peroxisome proliferator-activated receptor γ co-activator-1α; PPAR-γ: peroxisome proliferator-activated receptor γ; TGF-β: transforming growth factor-β; TIMP-1: tissue inhibitor of metalloproteinase 1; VSMCs: vascular smooth muscle cells.

Figure 2 summarizes the major biological effects of TRAIL on a variety of tissues which are pathogenetically involved in the development of diabetes, as demonstrated in animal and in vitro mechanistic studies.

## 7. The role of TRAIL in Diabetes-Related Complications

### 7.1. Atherosclerotic Cardiovascular Disease (ACVD)

The theory that TRAIL plays a functional role in the progression of ACVD is based on the direct effects of TRAIL on endothelial and vascular smooth muscle cells (VSMCs), which are both important for maintaining vascular homeostasis. This theory is further strengthened by the finding that TRAIL expression is reduced in the heart of diabetic dyslipidemic mice [78]. TRAIL has been shown to promote the proliferation of endothelial cells via activation of Akt/ERK pathways [8]; promote the survival, proliferation, and migration of VSMCs via activation of the ERK pathway [7]; and also increase the phosphorylation of endothelial nitric oxide synthase (eNOS) leading to an increased NO production and improved endothelial function [79]. Interleukin-18 (IL-18), which is commonly elevated in the setting of ACVD, has been recognized as an important negative regulator of TRAIL system leading to suppressed TRAIL expression by monocytes, which are thought to be the primary source of TRAIL production and secretion in the healthy circulation [80]. TRAIL suppression may in turn modulate the function of monocytes and macrophages in the direction of promoting inflammation and atherosclerosis [80]. Overall, possible mechanisms underlying the anti-atherosclerotic and vasoprotective effects of TRAIL in CVD include improved endothelial function [30], atherosclerotic plaque-stabilizing effects by increasing the number and migration of VSMCs [7,81], suppressive effects on vascular inflammation by modulating the phenotype and function of monocytes/macrophages [80], inhibitive effects on calcium-induced vascular calcification via modulation of the receptor activator of nuclear factor κΒ ligand (RANKL) [82], and cardioprotective effects by reducing cardiac fibrosis and adverse cardiac remodeling as shown in a mouse model of diabetic cardiomyopathy [83].

The major clinical manifestations of ACVD comprise coronary artery disease (CAD), cerebrovascular disease (stroke) and peripheral artery disease (PAD).

TRAIL is present in coronary atherosclerotic plaques in humans. Its expression is higher in vulnerable (rupture-prone) than stable atherosclerotic lesions and is induced by oxidized low density lipoprotein cholesterol (oxLDL) [26]. Circulating TRAIL levels have been associated with both the volume and qualitative composition of coronary plaques in humans [84]. In a study using intravascular ultrasound (IVUS) combined with virtual histology to identify vulnerable plaques, low serum TRAIL levels were associated with high-risk characteristics such as an increased necrotic core and a thin fibrous cap, suggesting a role of TRAIL in the maintenance of atherosclerotic plaque integrity [84]. Soluble serum TRAIL levels have been found to be reduced in patients with acute coronary syndromes such as acute myocardial infarction [25] or unstable angina [26] compared to patients with stable CAD or healthy individuals, and inversely correlated with serum inflammatory markers such as C-reactive protein (CRP) [26]. Interestingly, reduced serum TRAIL levels in patients with acute myocardial infarction may also have prognostic implications, since they were able to predict adverse outcomes such as death and heart failure one year after diagnosis, independently of traditional CVD risk factors [25]. The prognostic role of TRAIL is further complemented by the finding that low serum TRAIL levels can predict all-cause and CVD mortality over a follow-up period of six years in older community-dwelling subjects with prevalent CVD [85]. Especially in the setting of diabetes, the protective role of TRAIL against diabetes-related atherosclerosis was demonstrated more than a decade ago in apolipoprotein E knockout mice ApoE (−/−) which were used as an experimental model of atherosclerosis [17,81]. In these mice, TRAIL genetic deficiency on the background of a HFD induced a diabetic phenotype characterized by obesity, impaired glucose tolerance, insulinopenia, pancreatic islet inflammation, and dyslipidemia, and exacerbated the preexisting atherosclerosis by destabilizing atherosclerotic plaques [17]. In the same model of diabetic atherosclerotic mice, it was also shown that systemic TRAIL delivery stabilized atherosclerotic plaques by increasing their VSMC content, preserving the endothelial coverage and inducing apoptosis in macrophages infiltrating the vascular wall [81]. Another interesting piece of evidence provided in a large-scale Swedish epidemiological study is that patients with T2DM display elevated plasma levels of soluble TRAIL-R2 serving as a surrogate marker of endogenous TRAIL effect neutralization [86]. This soluble death receptor is released by peripheral blood mononuclear cells and pancreatic β-cells under conditions of metabolic stress leading to FasL-mediated apoptosis [86]. After adjustment for CVD risk factors in multivariate regression models, elevated soluble TRAIL-R2 levels were found to increase the risk of developing T2DM and CVD (death, myocardial infarction and stroke) over a period of 20 years [86]. These data highlight the potential role of soluble TRAIL death receptors as biomarkers of β-cell dysfunction and vascular injury, and as independent predictors of T2DM and CVD.

Similar findings have been reported for the role of TRAIL in cerebrovascular disease and PAD. Reduced serum-soluble TRAIL levels have been associated with an increased severity of acute ischemic stroke and an increased stroke volume in humans, without any difference between different stroke subtypes [87]. With regard to PAD, the role of TRAIL seems promising as well. A significant down-regulation of serum TRAIL levels has been reported in Korean patients with PAD and vascular calcification [88]. Animal data also suggest that TRAIL promotes angiogenesis after hindlimb ischemia in vivo [89]. TRAIL-deficient mice displayed reduced ischemia-induced neovascularization, while adenoviral TRAIL delivery improved limb perfusion and increased the capillary density and VSMC content [89]. These data highlight the potential of TRAIL to improve the angiogenic response to ischemia and increase perfusion recovery in diabetic patients with PAD.

### 7.2. Microvascular Complications

*Diabetic Nephropathy:* TNF superfamily members play an important role in renal pathophysiology by regulating a plethora of biological functions including cell proliferation, differentiation, apoptosis, necrosis, autophagy, inflammation, angiogenesis, and fibrosis in different etiologies and stages of kidney disease [90]. Under normal conditions, TRAIL is expressed mainly by the renal tubules, and less by the glomeruli [91].

In vitro studies have shown that TRAIL exerts proapoptotic effects on renal tubular cells exposed concomitantly to hyperglycemia and proinflammatory cytokines; therefore, it has been postulated that TRAIL-induced apoptosis might contribute to the pathophysiology of diabetic nephropathy [91].

In a mouse model of T2DM, TRAIL deficiency was found to promote diabetic nephropathy, since TRAIL-deficient mice exhibited increased urinary protein loss and more severe glomerular damage compared with wild-type mice [92]. These data are further strengthened by another experimental study in a mouse model of obesity and T2DM (db/db mice) complicated by diabetic nephropathy, which unraveled the renoprotective effect of therapeutic TRAIL administration in severe forms of diabetic disease [34]. This study could show that TRAIL treatment improved renal function without affecting proteinuria, reversed the abnormal glomerular and tubular morphology associated with diabetic nephropathy, and prevented TGF-β (transforming growth factor-β)-mediated renal fibrosis through mechanisms possibly related to suppression of inflammation and rescue of autophagy [34]. These anti-fibrotic and renoprotective effects were mediated by DR5, and were independent of glucose control [34].

In humans, reduced circulating TRAIL levels have been reported in several cohorts of T2DM patients with diabetic nephropathy compared with healthy individuals [27,28,57]. In 112 patients with T2DM, the presence of microalbuminuria was associated with lower serum TRAIL levels compared to non-diabetic controls [57]. Similarly, serum-soluble TRAIL levels were found to be significantly suppressed in a cohort of 22 insulin-treated T2DM patients with diabetic nephropathy expressed as macroalbuminuria and foot ulcers [27]. In line with these findings, both soluble TRAIL levels and TRAIL mRNA expression in peripheral blood mononuclear cells were significantly reduced in Chinese patients with diabetic nephropathy in parallel with a significant increase in circulating inflammatory markers such as interleukin-1 (IL-1), IL-6, TNF-α and monocyte chemoattractant protein-1 (MCP-1) [28]. Of note, renal TRAIL expression has been found to be increased in renal biopsies of patients with diabetic nephropathy, in correlation with the clinical and histological severity of the disease, consistent with the theory of increased TRAIL consumption at sites of active inflammation [91].

Although the above experimental and clinical data are definitely interesting since they shed light on the putative modulating role of TRAIL for diabetes-related organ damage, given the limited number of studies in this field, the role of TRAIL in the development and progression of diabetic nephropathy and its potential prognostic and therapeutical implications warrant consolidation in future studies.

*Diabetic Retinopathy:* The detection of extremely high levels of soluble TRAIL in the conjunctival sac of the anterior surface of the eye, compared to other body fluids, has important implications for maintaining the immune surveillance of the eye, and has inspired the investigation of a possible role of TRAIL for the pathogenesis of diabetic ocular complications such as diabetic retinopathy and macular degeneration.

Proliferative diabetic retinopathy (PDR) is caused by widespread ischemia of the inner retinal layers, leading to new vessel formation. It is considered as a wound-healing process, characterized by neovascularization, inflammation, and fibrovascular contraction, leading to potentially sight-threatening complications such as hemorrhage, retinal detachment, and blindness [93]. Proangiogenic factors produced by the ischemic retina are thought to be the major causal factors implicated in the neovascularization process. It has been suggested that TRAIL might protect against PDR by restraining retinal endothelial cell proliferation. In support of this notion, TRAIL deficiency has been associated with a delayed regression of retinal neovascularization, and recombinant TRAIL has been shown to promote apoptosis of retinal endothelial cells in mice [94]. Extrapolating these findings to the clinical setting, soluble TRAIL levels in the conjunctival sac fluid [95] and in vitreous samples [29] have been found to be significantly decreased in patients with PDR. The anti-inflammatory and anti-angiogenic role of TRAIL, which is important to secure the anatomic and functional stability of the ocular surfaces, lends credence to the hypothesis that a decreased production and/or release of TRAIL might aggravate PDR by enhancing inflammation and reducing the degree of apoptosis in retinal endothelial cells.

TRAIL-induced apoptosis has been also implicated in age-related macular degeneration (AMD), an important ocular disorder representing a leading cause of irreversible vision loss in the elderly, especially in the setting of diabetes. Interestingly, lower levels of TRAIL-R3 have been documented in serum samples of patients affected by AMD compared to control subjects [96]. The authors suggested that the low levels of TRAIL-R3 in these patients may actually increase the amount of TRAIL interacting with the proapoptotic receptors (TRAIL-R1 and TRAIL-R2), resulting in enhanced TRAIL-mediated apoptosis of photoreceptors and retinal pigment epithelial cells, which is an important contributor to the pathogenesis of AMD [97].

## 8. Summary and Concluding Remarks

There is no doubt that TRAIL represents a molecule of unique interest with versatile biology and complex mechanisms of action. It is distinguished from other TNF-related cytokines in that it can selectively induce either apoptosis or survival in a cell-type specific context depending on circulating concentrations, receptor expression and local stimuli. Beyond its anti-tumor properties, an accumulating body of experimental and clinical evidence over the past two decades suggests a protective role of TRAIL in the development of T1DM and T2DM as well as their complications. The concept of this protective role is strengthened by the following observations: (i) T1DM and T2DM are accelerated and exacerbated by TRAIL blockade or genetic deficiency (animal models), (ii) A broad spectrum of metabolic abnormalities associated with T1DM and T2DM can be prevented and ameliorated with TRAIL treatment or systemic TRAIL gene delivery (animal models), (iii) circulating serum soluble TRAIL levels are significantly reduced in patients with T1DM and T2DM both at onset and in more advanced stages of diabetes-related complications such as atherosclerotic cardiovascular disease and diabetic nephropathy, (iv) serum TRAIL levels progressively increase upon antidiabetic treatment, (v) serum levels of soluble TRAIL-R2 are elevated in diabetic patients and serve as a biomarker with independent predictive utility for the occurrence of T2DM and CVD in non-diabetic individuals. Animal and in vitro studies have reported direct biological actions of TRAIL on multiple tissues involved in diabetes pathophysiology including pancreatic islets, skeletal muscle, adipose tissue, liver, kidney, immune and vascular cells.

The major suggested mechanisms underlying the protective role of TRAIL in T1DM involve its direct inhibitory effects on autoreactive immune cells infiltrating pancreatic islets; the suppression of proinflammatory cytokine signaling; the inhibition of pancreatic matrix degradation, which is thought to prevent cytokine-induced insulitis; and finally, the inhibition of apoptosis and stimulation of proliferation of pancreatic β-cells. The beneficial effects of TRAIL in the setting of T2DM have been mainly attributed to its immunosuppressive properties which counteract inflammation, proliferative effects on pancreatic β-cell mass, insulin-sensitizing and myogenic effects on skeletal muscle, and protective effects on liver consisting in NAFLD amelioration. The possible mechanisms underlying the anti-atherosclerotic and vasoprotective effects of TRAIL in the setting of CVD include improved endothelial function, atherosclerotic plaque-stabilizing effects, suppressive effects on vascular inflammation, inhibitive effects on vascular calcification, and cardioprotective effects achieved by reducing cardiac fibrosis and adverse cardiac remodeling.

Although there is substantial evidence suggesting an important role of TRAIL for the natural course of T1DM and T2DM, the emerging TRAIL-diabetes relationship is not without controversies and open questions. TRAIL and its receptors are expressed in a wide variety of tissues, but the primary cellular source of circulating TRAIL and the precise mechanisms regulating TRAIL secretion remain incompletely understood. Detailed studies quantifying TRAIL expression in different cell subpopulations are therefore of special interest. There is also still residual controversy regarding the effect of TRAIL on fat cell metabolism, since both positive (adipose tissue expandability) and negative (inflammation and insulin resistance) effects have been reported. Possible explanations for the conflicting data on the effects of TRAIL on adipose tissue may be related to the experimental models of obesity studied, issues of species- and tissue-specificity, and most importantly the limited capacity of in vitro experiments to capture the complex crosstalk of adipose tissue with other tissues and accurately reflect the in vivo physiology.

Although the existing evidence is sufficient to inspire the investigation of TRAIL-related interventions (TRAIL gene therapy, recombinant TRAIL treatment, TRAIL receptor agonists) as a strategy to improve metabolic risk factors in diabetic patients, especially considering the acceptable safety profile demonstrated in clinical trials testing TRAIL in cancer patients, caution is warranted in the extrapolation of animal and in vitro data to the clinical setting. Further research in humans is imperative in order to fully elucidate all the aspects of the complex TRAIL-diabetes relationship. Future research in humans should address the course of circulating soluble TRAIL levels in diabetic patients at different stages of the disease and in the presence of different diabetes-related comorbidities, and explore the impact of glycemic control and antidiabetic treatment on TRAIL alterations.

## Figures and Tables

**Table 1:** A summary of the major experimental and clinical studies investigating the relationship of TRAIL with T1DM in animal models and humans.

First Author (Year of Publication)	Experimental Model or Study Population	TRAIL-Related Intervention (If Applicable)	Study Methods	Key Findings
Animal data				
Lamhamedi-Cherradi (2003)	NOD mice challenged with cyclophosphamide Normal and TRAIL-deficient C57BL/6 mice treated with multiple low doses of streptozotocin	Soluble TRAIL receptor (sDR5) to block TRAIL function TRAIL gene knockout	Induction of diabetes, production of recombinant human sDR5, ELISA, histochemistry, quantification of islet inflammatory lesions, cell cultures, analyses of cell viability and apoptosis	Accelerated diabetes onset ↑ severity of autoimmune insulitis in pancreatic islets ↑ GAD65-specific immune responses ↑ incidence and extent of islet inflammation in TRAIL-deficient mice
Mi (2003)	NOD mice challenged with cyclophosphamide NOD mice receiving diabetogenic spleen T-cells from newly-diagnosed diabetic NOD mice	Soluble TRAIL receptor (sDR5) to block TRAIL function	Induction of diabetes, production of recombinant human sDR5, splenic T-cell isolation and proliferation assays, T-cell adoptive transfer, cell cultures, gene expression profiling of pancreatic islets, analyses of cell viability and apoptosis, ELISA, immunoblotting	↑ incidence of cyclophosphamide-induced T1DM ↑ incidence and earlier onset of T1DM post-transfer of diabetogenic T-cells
Dirice (2009)	Rats treated with multiple low doses of streptozotocin	Adenovirus-mediated TRAIL gene delivery into pancreatic islets (Ad5hTRAIL)	Ex vivo genetic engineering of pancreatic β-cells, transplantation of genetically modified pancreatic islets in streptozotocin-induced diabetic rats, metabolic assays, ELISA, pancreas histology	Prolonged normoglycemia ↓ severity of insulitis Extended islet graft survival and function
Zauli (2010)	C57BL/6 mice treated with multiple low doses of streptozotocin	Recombinant TRAIL treatment (intraperitoneal injections) for 5 days In vitro exposure of human/mouse PBMCs and isolated human islets to recombinant TRAIL	Islet isolation, cell cultures, RNA and protein analyses, metabolic assays, ELISA, pancreas histology	↓ hyperglycemia ↑ body weight ↑ insulin secretion Partially preserved islet morphology and function ↓ TNF-α, ↓ OPG, ↓ VCAM-1 expression in TRAIL-treated mice ↑ SOCS1 expression in PBMCs and human islets exposed in vitro to TRAIL
Kang (2010)	NOD mice	Adenovirus-mediated systemic human TRAIL gene delivery (iv injection)	Metabolic assays, cell cultures, RNA extraction and RT-PCR in pancreas and liver, pancreatic islet isolation and histopathological analysis, cell viability and flow cytometry apoptosis assays, Western blot analysis, ELISA for plasma cytokine and TIMP-1 measurements, gelatin zymography for the inhibition of MMPs	↓ hyperglycemia ↑ TIMP-1 expression ↓ pancreatic MMP activity ↓ cytokine-induced insulitis and apoptosis Prevention of T1DM development
**Clinical data**				
Tornese (2014)	507 pediatric subjects n = 387 patients with T1DM n = 98 healthy controls n = 22 healthy AA-positive subjects	NA	Retrospective study ELISA for serum soluble TRAIL measurements	↓ serum soluble TRAIL levels in T1DM vs. other groups ↓ serum soluble TRAIL levels in T1DM patients presenting with DKA at onset (vs. those without DKA) Inverse correlation between serum TRAIL levels at diagnosis and insulin requirements up to 2 years of follow-up
Tornese (2015)	n = 11 pediatric patients with newly diagnosed T1DM complicated by DKA and secondary DKA	NA	Pilot study ELISA for serum soluble TRAIL measurements at sequential time points after admission, blood gas analysis for metabolic status assessment	↑ serum soluble TRAIL levels shortly after insulin administration and metabolic stabilization Inverse correlation between serum TRAIL levels and the degree of metabolic decompensation

Abbreviations: AA-positive: autoantibody-positive; DKA: diabetic ketoacidosis; ELISA: enzyme-linked immunosorbent assay; GAD65: glutamic acid decarboxylase 65; iv: intravenous; MMPs: matrix metalloproteinases; NA: not applicable; NOD: non-obese diabetic; OPG: osteoprotegerin; PBMCs: peripheral blood mononuclear cells; RT-PCR: reverse transcriptase polymerase chain reaction; sDR5: soluble death receptor 5; SOCS1: suppressor of cytokine signaling 1; T1DM: type 1 diabetes mellitus; T2DM: type 2 diabetes mellitus; TIMP-1: tissue inhibitor of metalloproteinase 1; TNF-α: tumor necrosis factor-α; TRAIL: tumor necrosis factor-related apoptosis-inducing ligand; VCAM-1: vascular cellular adhesion molecule-1.

**Table 2:** A summary of the major experimental and clinical studies investigating the relationship of TRAIL with T2DM in animal models and humans.

First Author (Year of Publication)	Experimental Model or Study Population	TRAIL-Related Intervention (If Applicable)	Study Methods	Key Findings
Animal data				
Di Bartolo (2011)	ApoE (−/−) HFD-fed mice	TRAIL gene knockout	Metabolic assays, RNA extraction and gene expression analysis, pancreatic islet histology, immunohistochemistry, morphometric analysis of atherosclerotic plaques	↑ body weight ↑ glycemia ↓ insulinemia ↓ islet insulin ↑ serum lipids ↑ pancreatic islet inflammation/apoptosis IGT Β-cell dysfunction Exacerbated atherosclerosis and plaque instability
Bernardi (2012)	HFD-fed C57BL/6 mice	Weekly intraperitoneal injections of recombinant human TRAIL for 12 weeks	Metabolic assays, gene expression analysis in adipose tissue, ELISA for cytokine measurements	↓ weight gain ↓ hyperglycemia ↓ hyperinsulinemia ↑ peripheral insulin sensitivity ↑ SM FFA oxidation ↓ proinflammatory cytokines ↓ adipogenic gene expression
Cartland (2017)	HFD-fed mice n = 9 healthy humans n = 10 obese patients n = 10 patients with hepatic steatosis n = 10 patients with NASH	TRAIL gene knockout	Plasma biochemistry, glucose and insulin tolerance tests, ex vivo glucose uptake studies, liver histology, tissue cultures, RNA extraction and RT-PCR for gene expression analysis, protein extraction and Western blotting, ELISA for serum soluble TRAIL measurements	In TRAIL-deficient mice: ↑ plasma lipids ↑ plasma glucose and insulin levels ↑ systemic insulin resistance ↓ Akt phosphorylation, GLUT4 expression and glucose uptake in SM ↑ hepatic steatosis, inflammation and fibrosis ↑ hepatic gene expression related to lipogenesis and gluconeogenesis ↑ expression of proinflammatory cytokines In patients with NASH: ↓ serum soluble TRAIL levels (vs. steatosis and obese)
Bernardi (2018)	HFD-fed C57BL/6 mice	Weekly injections of recombinant human TRAIL for 8 weeks	Metabolic assays, tissue collection and histology, in vitro studies on HepG2 cells and mouse primary hepatocytes	↓ body weight ↓ adipocyte hypertrophy ↓ FFAs ↓ inflammatory markers ↓ liver fat content ↑ hepatic PGC-1α expression Improved IGT Improved NAFLD
Toffoli (2021)	HFD-fed C57BL/6 and db/db mice	Intraperitoneal injections of recombinant human TRAIL for 8–12 weeks	Production of recombinant human TRAIL, SM extraction (quadriceps), glucose uptake studies, FFA oxidation experiments, gene expression quantification by RT-PCR, DR5 silencing, immunofluorescence, Western blot analysis, histology + in vitro studies on mouse C2C12 myoblasts	Effects on SM: ↑ Akt phosphorylation ↑ insulin-stimulated glucose uptake ↑ myofiber size ↑ myogenin and PGC-1α expression ↑ myogenesis (muscle differentiation) No effect on lipid accumulation in skeletal myotubes
**Clinical data**				
Bisgin (2012)	n = 22 newly diagnosed drug-naïve patients with T2DM	NA	ELISA for serum soluble TRAIL measurements	↓ serum soluble TRAIL levels in T2DM patients (vs. controls)
Arik (2013)	n = 22 insulin-treated patients with T2DM, DN (macroalbuminuria) and foot ulcers	NA	ELISA for serum soluble TRAIL measurements	↓ serum soluble TRAIL levels in patients with DN and foot ulcers (vs. non-diabetic controls) No correlation between serum TRAIL levels and HbA1c or fasting glucose levels
Xiang (2014)	n = 55 newly diagnosed patients with T2DM	NA	ELISA for serum soluble TRAIL measurements	↓ serum soluble TRAIL levels in T2DM patients (vs. non-diabetic controls) ↑ serum soluble TRAIL levels after 6 months of antidiabetic treatment Absolute change in serum TRAIL levels ~ absolute change in HbA1c, fasting and postprandial glycemia before and after treatment
Chang (2018)	n = 42 patients with T2DM n = 42 patients with DN n = 42 healthy controls	NA	Real-time RT-PCR for TRAIL mRNA levels in PBMCs ELISA for serum cytokine and TRAIL measurements	↓ TRAIL mRNA in PBMCs and ↓ serum soluble TRAIL levels in patients with T2DM and DN (vs. controls) ↑ proinflammatory cytokines in patients with DN (vs. controls)
Choi (2018)	n = 112 patients with T2DM	NA	ELISA for serum soluble TRAIL measurements	↓ serum soluble TRAIL levels in T2DM patients with microalbuminuria (vs. controls) Inverse correlation between serum TRAIL levels and HbA1c

Abbreviations: Akt: protein kinase B; ApoE (−/−): apolipoprotein E knockout; db/db: leptin receptor-deficient mice; DN: diabetic nephropathy; DR5: death receptor 5; ELISA: enzyme-linked immunosorbent assay; FFA: free fatty acid; GLUT4: glucose transporter 4; HbA1c: glycated hemoglobin A1c; HepG2: hepatoma G2; HFD: high fat diet; IGT: impaired glucose tolerance; NA: not applicable; NAFLD: non-alcoholic fatty liver disease; NASH: non-alcoholic steatohepatitis; PBMCs: peripheral blood mononuclear cells; PGC-1α: peroxisome proliferator-activated receptor γ co-activator-1α; RT-PCR: reverse transcriptase polymerase chain reaction; SM: skeletal muscle; T2DM: type 2 diabetes mellitus; TRAIL: tumor necrosis factor-related apoptosis-inducing ligand.

## Abbreviations

AA-positive: autoantibody-positive; ACVD: atherosclerotic cardiovascular disease; Akt: protein kinase B; AMD: age-related macular degeneration; ApoE (−/−): apolipoprotein E knockout; CAD: coronary artery disease; CD95L: cluster of differentiation 95L; CRP: C-reactive protein; CVD: cardiovascular disease; db/db: leptin receptor-deficient mice; DcR1: decoy receptor 1; DcR2: decoy receptor 2; DISC: death-inducing signaling complex; DKA: diabetic ketoacidosis; DN: diabetic nephropathy; DR4: death receptor 4; DR5: death receptor 5; ECs: endothelial cells; ELISA: enzyme-linked immunosorbent assay; eNOS: endothelial nitric oxide synthase; ERK1/2: extracellular signal regulated kinases 1 and 2; FasL: Fas Ligand; FFA: free fatty acid; GAD65: glutamic acid decarboxylase 65; GLUT4: glucose transporter 4; HbA1c: glycated hemoglobin A1c; HepG2: hepatoma G2; HFD: high fat diet; IGT: impaired glucose tolerance; IL-1: interleukin-1; IL-2: interleukin-2; IL-4: interleukin-4; IL-6: interleukin-6; IL-18: interleukin-18; INF-γ: interferon-γ; iv: intravenous; IVUS: intravascular ultrasound; JNK: c-Jun N-terminal kinase; MAPK: mitogen-activated protein kinase; MCP-1: monocyte chemoattractant protein-1; MMPs: matrix metalloproteinases; NA: not applicable; NAFLD: non-alcoholic fatty liver disease; NASH: non-alcoholic steatohepatitis; NF-κB: nuclear factor kappa B; NK cells: natural killer cells; NO: nitric oxide; NOD: non-obese diabetic; OPG: osteoprotegerin; oxLDL: oxidized low density lipoprotein cholesterol; PAD: peripheral artery disease; PBMCs: peripheral blood mononuclear cells; PDR: proliferative diabetic retinopathy; PGC-1α: peroxisome proliferator-activated receptor γ co-activator-1α; PI3K: phosphatidyl-inositol 3 kinase; PPAR-γ: peroxisome proliferator-activated receptor γ; RANKL: receptor activator of nuclear factor κΒ ligand; RT-PCR: reverse transcriptase polymerase chain reaction; sDR5: soluble death receptor 5; SM: skeletal muscle; SOCS1: suppressor of cytokine signaling 1; T1DM: type 1 diabetes mellitus; T2DM: type 2 diabetes mellitus; TGF-β: transforming growth factor-β; TIMP-1: tissue inhibitor of metalloproteinase 1; TNF-α: tumor necrosis factor-α; TNF-SF10: TNF superfamily member 10; TRAIL: tumor necrosis factor-related apoptosis-inducing ligand; TRAIL-R1: TRAIL receptor 1; TRAIL-R2: TRAIL receptor 2; TRAIL-R3: TRAIL receptor 3; TRAIL-R4: TRAIL receptor 4; TRAIL-R5: TRAIL receptor 5; Treg: regulatory T-cells; VCAM-1: vascular cellular adhesion molecule-1; VSMCs: vascular smooth muscle cells.
